# Rethinking progress: harmonizing the discourse on genetically modified crops

**DOI:** 10.3389/fpls.2025.1547928

**Published:** 2025-03-21

**Authors:** Abreham Bekele-Alemu, Obssi Dessalegn-Hora, Tura Safawo-Jarso, Ayalew Ligaba-Osena

**Affiliations:** ^1^ Laboratory of Plant Molecular Biology and Biotechnology, Department of Biology, University of North Carolina Greensboro, Greensboro, NC, United States; ^2^ Department of Biology, College of Natural Sciences, Salale University, Fiche, Ethiopia; ^3^ Ethiopian Agricultural Research Institute, National Agricultural Biotechnology Research Center, Addis Ababa, Ethiopia

**Keywords:** GM crops, biotech crops, IPRs, safety concerns, terminator technology

## Abstract

Genetically modified crops (GM crops) also known as biotech crops are crops that have been altered through genetic engineering techniques and under cultivation for approximately 28 years. By October 2024, over 30 nations have approved the cultivation of GM crops. The global area utilized for biotech crop production has reached 206.3 million hectares. Despite the substantial growth in the cultivation of these crops, debate continues between proponents and opponents of GM crops. In this article, critical concerns and common ground between the arguments of both sides were described. The main issues addressed include the naturalness of GM crops, religious perspectives, beneficial aspects, safety issues, socio-economic impacts and intellectual property rights. We argue that the classification GM crops as unnatural is a claim that lacks scientific reality. In a similar vein, comparing GM technology to the act of playing God is inappropriate. Moreover, the belief that GM crops do not contribute to yield improvements is inconsistent with empirical evidence. Additionally, the claim that foods produced from GM crops are unsafe for human consumption holds unseen concerns that is not on the ground. We have also highlighted the necessity of implementing intellectual property rights that support seed developers for a limited duration without violating farmers’ rights. In conclusion, as a consumer has the right to know what they eat, labeling of GM food products fosters transparency and enhance consumer autonomy.

## Introduction

**Figure 1:**
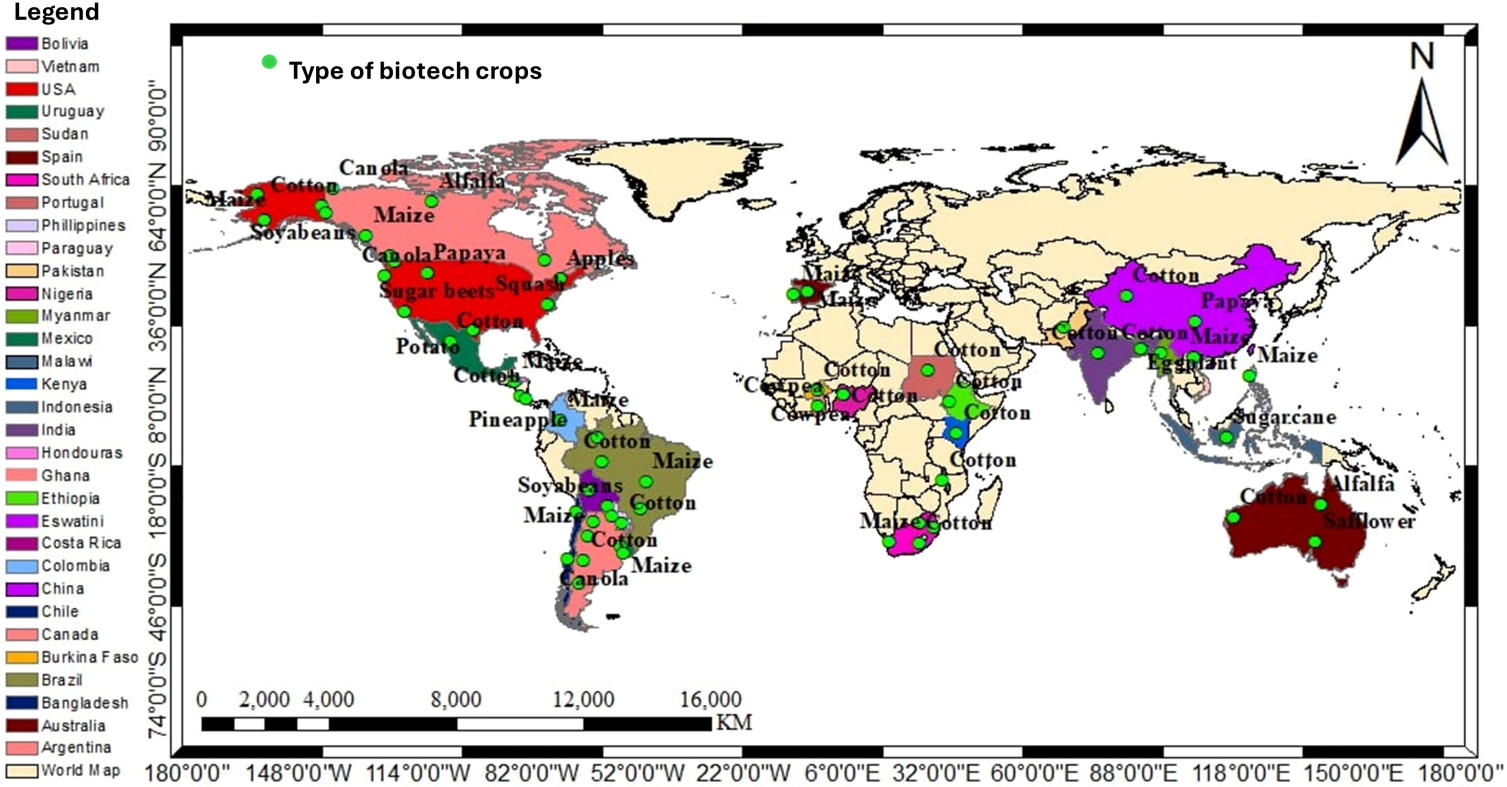
Global map of approved biotech crop(s) producing countries on farm in 2024.

Various definitions exist for genetically modified organisms (GMOs), however a more comprehensive definition for GMO is *‘an organism whose genome has been manipulated to enhance desired physiological traits or produce desired biological products’* (Fridovich-Keil and Diaz,., 2023). Flavr Savr tomato was the first GM crop developed in 1994 for delayed ripening and approved by the Food and Drug Administration (FDA) for marketing in the USA (Baranski et al., 2019). Subsequently, other transgenic crops such as canola with modified oil composition, *Bacillus thuringiensis* (Bt) corn/maize, Bt cotton, Bt potatoes, cotton resistant to the herbicide bromoxynil, glyphosate-resistant (GR) soybeans, and many other GM crops have also received marketing approval (Kamthan et al., 2016). The production of GM crops expanded every year, and in 2024 global land use for biotech crops reached 206.3 million hectares (AgbioInvestor, 2024) and about 32 countries so far granted cultivation approval (Shahbandeh, 2024) as indicated in **Figure 1**. This article reviews the strategies employed to develop GM crops, existing gap in scientific communication, public concerns, and safety measures, labelling issues, intellectual property rights.

Though it is relatively decreasing, the safety of genetically modified foods has been a topic of debate for the last three decades, with different viewpoints highlighting both advantages and drawbacks (Bawa and Anilakumar, 2013; Evanega et al., 2022). Utilization of GMOs has initiated various discussions, with some people criticizing GMOs to be of no use and interfering with nature, while others claim that GM crops are not effective in boosting crop production to feed ever-increasing population and pose risks to human health (Raman, 2017). Debates are also ongoing regarding GM product labeling, unnecessary monopolization of seeds, and the introduction of infertile traits to seeds (Kim et al., 2022). In this article, we addressed the major arguments claiming GMOs as unnatural entity and playing God, the claim that GM crops do not produce better yields, and that they are unsafe, and several other concerns of public interest regarding GM crops. After addressing some of the most common arguments, we propose a way to benefit from biotechnological advancements while maintaining moral and ethical values based on scientific realities. Furthermore, we are summarizing the pros and cos related to intellectual property rights; second generation infertile seeds by considering the concerns of both sides.

## The need for rational discourse on GM crop controversies

Since the release of GM crops, there have been controversies that necessitate engaging in logical discussions (Ghimire et al., 2023; Tahir et al., 2024). Consumers often find themselves uncertain and confused whether to consume foods derived from GM crops (Blagoevska et al., 2021). Through the facilitation of rational discussions, individuals can engage in a critical analysis regarding GM crops from diverse viewpoints, thereby enhancing their understanding of complex issues (Marzinkowski and Engelmann, 2022). Rationalization serves as a crucial mechanism for facilitating informed decision-making by employing a scientific assessment of risks and benefits grounded in empirical evidence. This process enhances transparency, mitigates concerns, and shapes public perception. By relying on evidence, ethical considerations, and the societal implications of their choices, stakeholders can move beyond speculation (Hielscher et al., 2016; Evanega et al., 2022). Moreover, rational debates enhance trust among scientists, policymakers, and the public by assessing the risks and benefits associated with GM crops and foods (Poot et al., 2018). Additionally, logical discussions provide a platform to address issues like ecological impacts, unintended consequences, and ethical dilemmas related to GM crops. Policymakers can leverage rational discourse to establish effective regulations that balance innovation and precaution (Abushal et al., 2021). By sharing accurate information through informed discussions, rationalizing debates helps correct misconceptions and shape public opinion.

Moreover, participating in rational discourse is instrumental in identifying logical fallacies, biases, and emotional appeals, thereby enabling individuals to make decisions grounded in robust evidence (Vrbová and Müllerová, 2021; Cusimano and Lombrozo, 2021). This process is essential for promoting reasoned and coherent conversations. By reducing emotional biases and focusing on verifiable data, rational discourse ensures that decisions are founded on logical reasoning rather than subjective beliefs (Chevallard, 2024). Furthermore, it promotes a more productive exchange of ideas, prompting participants to critically assess their arguments and explore alternative perspectives (Hielscher et al., 2016). Participating in rational debates not only enhances understanding of the topic at hand but also increases the likelihood of uncovering common ground or reaching a consensus. Additionally, the rationalization process aids in pinpointing fallacies and unsubstantiated claims, thereby encouraging more informed and thorough conversations.

## Public understanding of GM crops, foods thereof, and the information gap

There exists a notable disparity between the beliefs held by scientists and the public perceptions. The understanding of GM crops among the public varies significantly, ranging from individuals possessing a well-rounded knowledge of the scientific principles underpinning their development and potential advantages, to those with limited information or misconceptions (Cui and Shoemaker, 2018; Ortega et al., 2022). Caradus (2023) states that the public’s attitude is significantly influenced by the credibility and dependability of the information they receive, as well as whether it is based on factual evidence, opinion, or a deliberate attempt to deceive. In his study, Caradus (2023) distinguishes between two types of false information: misinformation, which is the unintentional dissemination of false information without any intention to cause harm, and disinformation, which involves the deliberate spread of false information with the aim of manipulating the truth and distorting facts through confusion. Consequently, the misunderstandings and criticisms surrounding GM crops and foods thereof can arise from both unintentional dissemination of false information and intentional efforts to mislead.

In a global context, public opinion on GM food is diverse, with a median of 48% of individuals across 20 countries considering GM foods unsafe, while only 13% regard them as safe (Funk et al., 2020). A substantial proportion, with a median of 37%, expresses a lack of knowledge. Notably, in countries like Russia (70%), Italy (62%), India (58%), and South Korea (57%), most of the population perceives GM foods as generally unsafe for consumption. This survey results indicate a significant deficiency in information and expertise concerning GM crops/foods. Strobbe et al. (2023) reported that individuals’ viewpoints on biotech crops and foods are commonly influenced by unfavorable assessments of their risk benefits and perceived lack of naturalness. Thus, it is very crucial to disseminate precise and unbiased information regarding GM crops to empower the public to make well-informed choices and engage in substantial dialogues concerning their utilization.

According to literature, significant disparities in understanding exist among study disciplines and countries. Studies have revealed that students pursuing technical and natural science programs tend to possess a better understanding of GM crops and their products compared to those enrolled in social sciences (Palmieri et al., 2020). Moreover, a considerable proportion of scientists specializing in Agricultural Science hold the belief that GM foods are safe for consumption, whereas the public was not found to share the same perspective (Wunderlich and Gatto, 2015; Pappalardo et al., 2021). The level of concern related to GM crops has shown to decline in Europe, decreasing from 63% in 2005 to 27% in 2019 (Ichim, 2021). This suggests an increasing level of understanding among the public regarding GM products. Through our examination and engagements with critics of GM crops, most of these individuals have a restricted comprehension of GM crops, viewing them exclusively as the exchange of genetic material across various species. Furthermore, they view GMOs as entirely novel synthetic entities in our food crops, originating from non-crop sources. Hence, it is imperative to provide a comprehensive understanding of GM technology development to facilitate a deeper comprehension of the various assertions.

Conversely, the lack of adequate understanding and differentiation among the various technologies employed in the development of GM crops leads to a further gap in information regarding GM crops and the foods derived from them. GM crops are developed through one of the three common methods: Transgenic, cisgenic, and subgenic (Sticklen, 2015). Transgenic crops are developed by transferring genes between unrelated species (Rajput et al., 2023). An example of this is crops engineered to have genes from bacterial species *Bacillus thuringiensis* (Bt) that helps to kill selected insects feeding this Bt-crops without having significant damage to other beneficial non target insects. The cisgenic modification involves manipulating genes within the same species (Venkataraman et al., 2019). This precise gene manipulation technique is far superior to non-precise conventional breeding methods. Cisgenic avoid non-important genes that may be co-integrated during conventional breeding (Schouten et al., 2006; Srinivas, 2023). Gene over-expression and transfer from wild type to breeding lines within the same species are typical examples of cisgenic crops. This targeted approach ensures that the desired traits are successfully transferred, resulting in more resilient, productive, and sustainable crops. However, even this precise extension of conventional breeding faces criticism from opponents of GM crops.

Another category of genetic modification is subgenic modification (Sticklen, 2015). This method involves the removal of non-important portions from the genome using advanced technologies such as CRISPR/Cas9 and RNA interference (Asmamaw and Zawdie, 2021; Aljabali et al., 2024; Bekele-Alemu et al., 2024). Unlike transgenic and cisgenic methods, subgenic modifications utilize splicing methods to precisely cut the host gene and eliminate unnecessary genes or unwanted RNA production. The CRISPR/Cas9 technology not only enables the removal of genes but also allows for the insertion, replacement, and knockout of genes (Ceasar et al., 2016; Adli, 2018; Bekele-Alemu et al., 2024).

Proponents of biotech crops argue that certain countries experienced a significant increase in agricultural output because of utilizing these biotech crops (AgbioInvestor, 2023). Despite the reported increase in production and overall advantages highlighted by advocates of biotechnology, an ongoing social and political dispute persists regarding the safety of GM crop-derived foods. According to proponents’ argument, over 3000 scientific studies and 284 institutions have evaluated the safety of these crops regarding human health and environmental consequences (Norero, 2022) which can guarantee the safety of biotech crops currently approved for production.

On the other hand, opponents argue that these biotech crops are “unnatural” and can cause significant harm to humans (Teferra, 2021). Approaching such claims involves consideration of two distinct perspectives. Firstly, it is important to recognize the presence of various safe synthetic substances used as medicinal remedies to protect human health from diseases (antibodies, antibiotics, vaccines and other of medicinal use). On the other hand, we must also accept the existence of anti-nutritional elements in our conventional crops, which can have negative impacts on our well-being, but human still rely on them. These elements include protease inhibitors, amylase inhibitors, lectins, tannins, phytic acid, gossypol, oxalates, cyanogens, saponins, nitrates, alkaloids, and anti-vitamins (Abu Hafsa et al., 2022). Therefore, the idea of naturalness does not always ensure the safety of a food, just as unnaturalness does not necessarily indicate harm. In the following sections, we will explore the major argument related to GM crops and their food products, highlighting their relationship with conventional breeds to bridge the gap between the current binary thinking.

## Are GM crops inherently unnatural?

As we mentioned earlier, one of the concerns raised by opponents of biotechnology is the idea of “unnaturality.” Anti-biotech activists and organic farmers consider GM crops as “unnatural.” (Verhoog, 2003). The belief that GMOs are not inherently natural has led to over 50 legal disputes where food companies have faced lawsuits for labeling their products as “natural” despite containing GM ingredients in the United States (Cerier, 2016). If we were told to consume foods with unnatural components, how many of us would like to do so? Assume that these foods contain uncommon amino acids in their protein and uncommon bases in their genome. We believe that no one would be able to consume such foods. The fear of unnaturality is a major topic of debate among anti-GMOs. On the other hand, supporters of biotech crops argue that these crops are not unnatural and that every food item has undergone some form of genetic modification since humans started crop domestication and breeding. Supporters claim that plant breeding techniques, such as cisgenic and subgenic strategies, are used to manipulate food crops through conventional breeding or natural mutagenesis (Telem et al., 2013; Cardi, 2016). Additionally, they argue that genes are naturally exchanged between plants and other organisms, making them equivalent to GMOs.

According to Lusk et al. (2014), the opposition towards GMOs frequently incorporates striking visual representations that exploit the availability heuristic and evoke associations with the fear of the unnatural within individuals’ memory. Additionally, it is worth noting the presence of the naturalistic fallacy which entails the belief that events occurring in nature are inherently positive, thereby leading to the negative perception of GMOs. In the current era of technology, individuals may easily fall into the trap of the naturalistic fallacy about their dietary decisions (Schultz and Morrison, 2014). It is crucial to bear in mind that the mere fact that a product is natural does not ensure its safety or superiority as a food choice. Biotech proponents often claim the naturalist debate as the means of clever marketing tactics, using it as propaganda to lean consumer opinions (Bovenkerk, 2012).

In her paper titled *‘Is Genetically Modified Food Unnatural*?’, Siipi (2015) argued that the terms natural and unnatural possess ambiguity. According to her point of view, specific interpretations of the term natural can provide valid justifications for preferring natural food over food that is deemed unnatural. The main argument put forth is that the term “natural” encompass the antithesis of the supernatural, autonomy from human intervention, appropriateness for sustenance, and compatibility with the environment. Siipi (2015) claimed that humans and other living beings are inherently natural and that the opposite of natural is the supernatural or anything that defies the laws of nature. She argues that GM food is as natural as its non-modified counterpart, as both are the result of natural processes. Accordingly, the concept of “natural” is often used to dismiss any moral concerns about unnatural things. Specifically, she believes that labeling GM food as unnatural is not a convincing argument for its moral suspicion.

Siipi’s argument raises interesting questions and presents compelling facts. The major question here is how does this unnatural phenomenon come into existence, and what are its fundamental building blocks? The exploitation of the dichotomy between natural and unnatural is frequently utilized to mislead consumers, thereby establishing a deceptive perception of superiority (Smith, 2019). When consumers are informed that a food item is unnatural, they anticipate the presence of toxic or unfamiliar chemicals that may pose a threat to human health. It is also important to understand that in nature, DNA consists of only four bases (A, T, C, and G) and twenty amino acids. We believe, any elements found in GM crops that deviate from these four bases and twenty amino acids should be deemed unnatural. This implies the existence of additional components beyond those found naturally. However, both GM and non-GM crops share the same fundamental constituents or building blocks. The only divergence, in certain cases, lies in the arrangement of these four bases and twenty amino acids. We also claim that the terms natural and unnatural are inadequate for distinguishing GM from non-GM crops. If critics of biotech crops contend that ‘unnatural’ encompasses anything altered by human intervention and ‘natural’ pertains to things untouched by human influence, it would logically imply that all the crops we currently consume, as well as the foods derived from them, are unnatural. This is because nearly all the food we ingest has undergone substantial modifications by nature and humans and can be deemed unnatural within this framework.

To enhance our understanding of the biochemistry involved in the digestive system, it is imperative to investigate whether long DNA molecules can be integrated into our genome and if large proteins play a direct role in muscle formation. However, it is essential to clarify that both hypotheses are unfounded. Upon ingestion, DNA is initially broken down into individual bases, which are then integrated into our genetic material during cell division according to their specific sequence in the genome. Similarly, large proteins are broken down by enzymes into individual amino acids, which are subsequently utilized in the synthesis of new proteins. Therefore, the distinction between natural and unnatural aspects of our food holds minimal significance and is overly ambitious. Nonetheless, it is important to recognize that certain natural proteins or other derivatives in our food items may pose potential risks prior to their breakdown and should not be deemed safe for human consumption. Given that GMO foods and crops undergo rigorous evaluation by various organizations before being approved for the market, crops with undesirable traits are less likely to be commercialized. In his publication entitled *‘Twenty-eight years of GM food and feed without harm: why not accept them*? Goodman (2024) reported that there have been no cases where post-market surveillance has identified harm to consumers or the environment, including the potential transfer of DNA from GM crops to non-target crops. This comprehensive review of twenty-eight years demonstrates that the arguments for restrictions on GM crops in certain countries, including developing nations, lack a rational basis, as there have been no legitimate safety concerns reported.

In an alternative perspective, critics of GM crops contend that these crops exhibit a lesser degree of naturalness when compared to traditional crops. This discourse primarily focuses on the dichotomy between “less natural” and “more natural.” As indicated in Siipi’s (2015) article, the term “less natural” is frequently linked with the notion of being “unnatural.” If one were to compare conventionally bred wheat or corn with their counterparts from a century ago, would they demonstrate uniform taste, nutritional content, size, and color? It is improbable that a positive response would be provided to these inquiries. Owing to the dynamic nature of our environment and the fluctuating levels of soil nutrients across time, the flavor profile, physical attributes, and elemental composition of these crops today markedly diverge from those 50 years ago. Nevertheless, opponents of GM crops continue to perceive conventionally bred crops as being more in harmony with nature, while viewing GM crops as less natural. From our perspective, the terminology of “natural versus unnatural” or “less natural versus more natural” lacks the requisite robustness to be applied definitively to GM crops.

Agronomist Norman Ernest Borlaug was one of the great scientists and is often known as the father of the green revolution of 1970s. Borlaug fronted global initiatives that greatly boosted agricultural production during the Green Revolution. In recognition of his efforts in developing high-yielding, semi-dwarf wheat varieties, Borlaug was awarded the Nobel Peace Prize in 1970. He is acknowledged for saving more than a billion individuals worldwide from famine, particularly in Mexico, Pakistan, and India. In this text, we are not to discuss the entirety of Borlaug’s remarkable contributions but rather to focus on his line of thoughts regarding GM crops in the early stages. Borlaug’s article titled *Ending World hunger: The promise of Biotechnology and the threat of antiscience zealotry* (Borlaug, 2000) provide a clear insight into his stance and arguments. Borloug claims that Neolithic humans domesticated nearly all food and animal species between 10,000 and 15,000 years ago. He claims that GM crops are not magic, but rather the gradual harnessing of nature’s forces for the advantage of feeding humanity, which began long before humans began altering crops through artificial selection. He considers artificial selection to be a genetic modification. He argues that the bread wheat we use today is the consequence of the hybridization of three separate plant genomes, each with a set of seven chromosomes, which can in principle fulfill the criteria of being GM crop. He further asserted that numerous generations of farmer descendants were subsequently accountable for effecting significant genetic alterations in all our primary crop and animal species and have not been treated differently than biotech crops.

Nowadays, many other scientists argue that modern food crops are not truly natural, citing examples like maize and wheat, which cannot exist without human intervention. While some GM crops involve human manipulation, others have undergone natural genetic changes without human involvement. For example, the T-DNA insertion in the F-box gene of sweet potato (Kyndt et al., 2015) is a form of natural modification. Similarly, transformations in the genus *Nicotiana* by *Agrobacterium rhizogenes* have played a significant role in its natural evolution (White et al., 1983; Suzuki et al., 2002). In 2016, researchers addressed the presence of genes from the *Agrobacterium* in cassava. These genes were believed to have been horizontally inserted approximately 8,000 years ago, contributing to the evolutionary development of the tuber into its present edible form (English, 2020). This report suggests that nature plays a crucial role in ensuring the well-being of organisms, ranging from bacteria to plants. Here one must clearly understand that being natural does not necessarily guarantee safety; and not all breeding processes are purely natural either.

In another study, scientists have recently made a significant discovery regarding monarch butterflies. It has been found that these butterflies have undergone genetic modifications due to the presence of viruses that specifically target Lepidoptera. These viruses have integrated their DNA into the genetic makeup of monarch butterflies throughout their evolutionary history (Gasmi et al., 2015). Consequently, it can be concluded that monarch butterflies possess foreign DNA, thus meeting the criteria for being genetically modified. Additionally, a recent investigation has uncovered that naturally occurring transgenic plant species, which are often referred to as GMOs, are more widespread than previously thought. Surprisingly, this list includes a variety of common food crops such as bananas, peanuts, cherries, hops, cranberries, and tea (English in 2020). These findings challenge the notion that GMOs are unnatural and highlight the need for a more nuanced understanding of genetic modification. Hence, it is crucial to acknowledge that categorizing GMOs as “unnatural” lacks logical justification and is equally deceptive.

**Figure 2:**
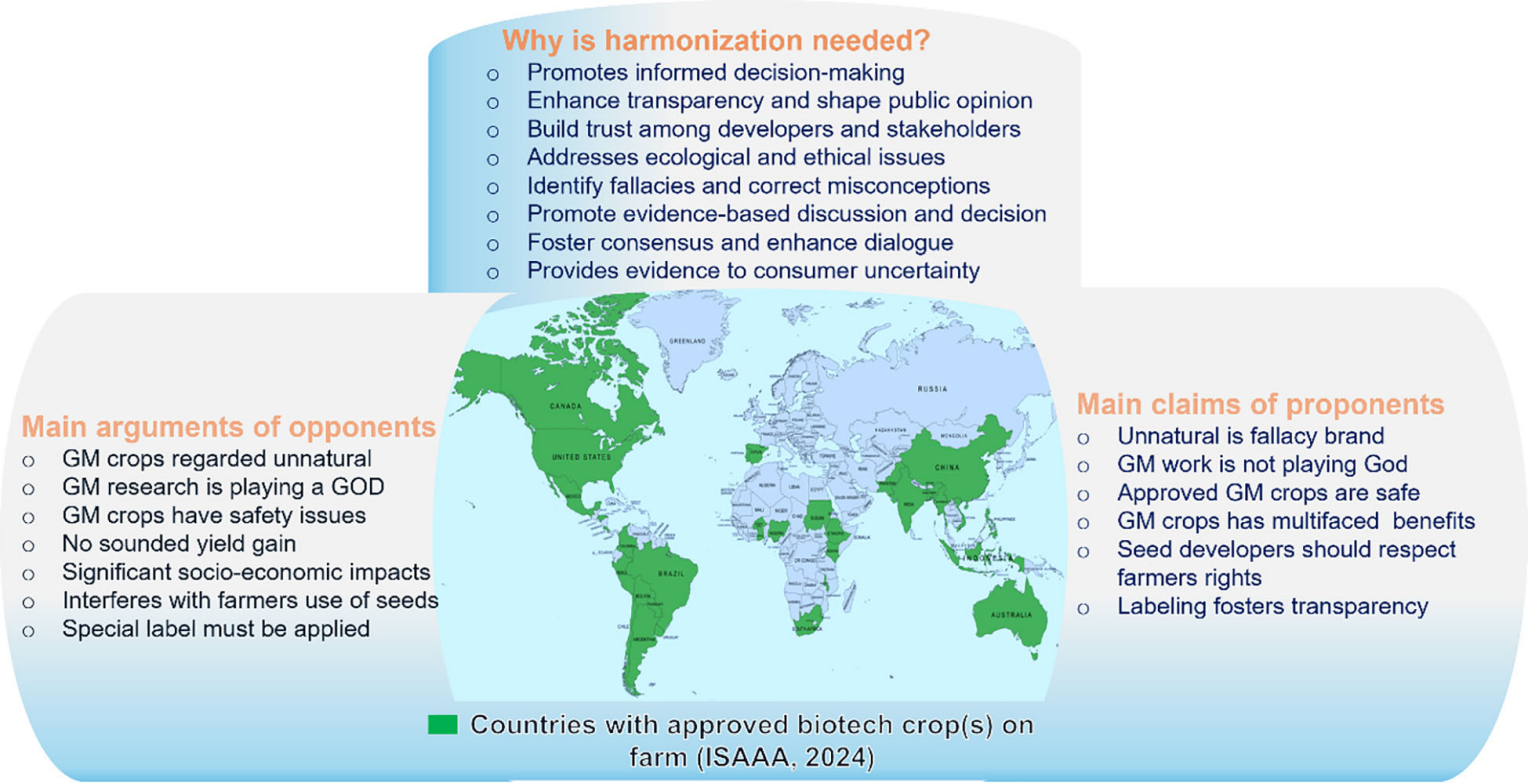
Major disparities between opponents and proponents and the need for harmonizing the debate regarding biotech crops. The central map shows countries with approved biotech crops.

In favor of GM crops, Borlaug contended that individuals with extreme views within the environmental movement are actively impeding scientific advancements. He asserted that when scientists align themselves with political movements that oppose scientific principles, the field of science loses its support base. Borlaug also questioned the hypothetical scenario of a world devoid of the technological progress achieved through GM crops. He ultimately concluded by asserting that GMO, being a swifter and more precise form of manipulation, serves as an extension of natural processes to produce crop varieties with enhanced yield and quality. According to Heldke (2015), proponents of GMO technology in the United States have been successful in gaining more acreage, despite facing opposition. This suggests that people are hesitant to consume foods that have been scientifically manipulated. If both sides fail to persuade each other, we believe the human population may suffer in terms of feeding the growing population. **Figure 2** summarizes the major disparities between opponents and proponents and the need for harmonizing the debate regarding biotech crops.

## Is GMO immoral and playing God?

The concept of immorality and its defiance against the divine is a topic that that frequently emerges in discussions related with GM foods and biotechnological advancements as part of conflict between religion and science (Degeling et al., 2014; Kalidasan and Das, 2022). According to Carter et al. (2021) ‘Playing God’ and ‘interfering with nature’ are prevalent intrinsic objections frequently raised against the biotechnological innovations. Playing God is a question that challenges our understanding of ethics and spirituality (Kirkham, 2006; Kalidasan and Das, 2022). The belief of what is deemed immoral and against the divine is a complex one, as it is shaped by cultural, religious, and personal beliefs. Ultimately, the pursuit of morality and righteousness is a journey that requires constant reflection and self-improvement.

The expression ‘playing God’ is frequently used by critics to contest the legitimacy of the use of GMO technology (Kotzé, 2016). These group argue that GM crops are morally objectionable and inherently unnatural. Conversely, some authors criticize that the notion of ‘playing God’ is irrelevant, fallacy grounded (Erde, 1989). From our viewpoint, dismissing GM crops as “playing God” is a regressive stance that opposes technological advancements and hinders progress. Throughout history, humanity has witnessed remarkable technological advancements that have revolutionized our lives. The invention of the airplane, the creation of the telephone, and the discovery of electricity innovations have propelled us forward into a new era. Some may argue that these achievements are analogous to playing God, but we believe that they are simply a testament to the incredible capabilities of the human mind.

According to Mehta (2001), genetic engineering of plants does not constitute “playing God” rather it is trust in science and suggested that religion and science need not be in conflict with each other. On the other hand, Omobowale et al. (2009) argue that the official stances of the three major monotheistic religions, namely Judaism, Islam, and Christianity, lean towards acceptance of GM technology. However, despite the official stances, there remains a knowledge gap and misunderstanding at the individual level in some locations. We also believe that it is natural to utilize our innate abilities to create and utilize technology. We argue that the development and use of technology should not be seen as playing God. Instead, it is a testament to our initiative and our desire to improve our live.

As Martin Heidegger once said (Wheeler, 2020), “Technology is not a means to an end; it is a way of revealing.” In other words, technology is not simply a tool that we use to achieve a particular goal; it is a way of uncovering new possibilities and revealing new aspects of the world around us. By embracing technology, we can expand our horizons and discover new ways of being in the world. Technology has allowed us to connect, to travel vast distances, and to access information at our fingertips. It has enhanced our understanding of the world and has the potential to address some of the greatest challenges we face as a species. Hence, it is important to recognize the immense value that technology brings to our lives.

It is important to recognize that religious beliefs and interpretations differ among individuals and faith traditions. While some may argue that altering the genetic makeup of organisms goes against the natural order established by a divine entity, others argue that humans have been granted the ability to manipulate and improve upon nature’s creations. From philosophical point of view, Kant’s moral philosophy emphasizes the importance of treating individuals as ends in themselves, rather than merely as means to an end (Johnson and Cureton, 2022). If we apply this principle to the context of GM crops, one could argue that the morality of genetic modification lies in the intentions and consequences behind its implementation. If GM crops are developed to benefit humanity, like boosting crop yields to reduce hunger, they could be considered morally justifiable and beneficial to people.

## Do GM crops outperform their non-GM counterpart in productivity?

Another ongoing discourse between those who oppose, and support biotech crops revolves around the question of whether the implementation of GM technology can truly enhance crop yield. Here, we are interested in mentioning some of the claim before two decades just to show how such claims can be propagative and affect public understanding at large. Altieri and Rosset (2002) presented some key arguments in their publication titled “Ten reasons why biotechnology cannot guarantee food security, environmental protection, and poverty reduction in the developing world,” wherein they voiced substantial concerns regarding the incapacity of GM technology to augment agricultural productivity. According to their early claims, the world already generates an ample amount of food per individual, thereby suggesting that there exists a sufficient food supply to nourish the global population and that much of the needed food can be produced by smallholder farmers located throughout the world using agroecological technologies. With more than 300 publications, Altieri is still claiming that agroecology is the best strategy for small farm sustainable development.

It is important to note that despite Altieri and Rosset (2002) early conclusion, hunger is still a major challenge affecting millions of people worldwide. In 2022 alone, nearly 800 million individuals worldwide experienced hunger, while 2.4 billion people lacked access to safe, nutritious, and adequate food (WHO, 2023). Moreover, the number of individuals facing acute food insecurity surged from 135 million in 53 countries before the COVID-19 pandemic to 345 million in 79 countries by 2023 (WFP, 2023). It has been suggested that to provide sustenance for the projected global population of almost 10 billion by 2050, there is a need to increase crop production by 50 to 70%. Meeting this challenge will likely require the development of new breeding and genetic engineering strategies (FAO, 2017). The claim posited by Altieri and Rosset (2002) concerning the achievement of food self-reliance can be perceived as a mere mirage, detached from the realm of tangible existence, and solely stemming from a reluctance to embrace biotechnology.

Another claim of Altieri and Rosset states that GM crops were not found to increase crop yield on the field trial, which we believe conveyed an immature conclusion at an early stage. Nevertheless, within the same vein, they acknowledge that Bt crops or herbicide-tolerant crops have exhibited yield increases ranging from 5% to 30%. On the other hand, GM proponents argue that the global area of transgenic crops has seen a remarkable increase to 206.3 million hectares in 2023 (AgbioInvestor, 2024). They emphasize that Altieri and Rosset overlooked a crucial aspect that GM crops serve diverse purposes, and not all crops are designed solely to enhance crop yield (Norero, 2018). Moreover, recent breakthroughs have demonstrated significant yield gains through the over-expression of specific genes. For example, Wei et al. (2022) reported a remarkable yield gain of 41-68% in rice and 17-23% in wheat by over-expressing a single gene in Chinese rice. Similarly, Fan et al. (2016) reported a 40% increase in rice yield through the overexpression of another single gene. Additionally, wheat with a 20% higher yield (Page, 2016), maize with a 25% yield increment (Pellegrino et al., 2018), and soybeans with 36% increase in production (Norero, 2018) were reported. It seems Altieri and Rosset are unable to secure victory over their opponents by any means afterward. According to Hansen and Wingender (2023), without the use of genetic modification, the world would have produced 33% less cotton,7% less maize, 5% less soybean and 2% less rapeseed in 2019. **Table 1** summarize yield gain (%) of nine approved GM crops currently grown on farmers’ field compared to the non-GM bred.

**Table 1:** Reported yield gain in nine approved GM crops currently grown on farmers’ field as compared to non-GM counterparts.

Type of crop	Major traits	Yield gain (%)	References
Soybean	Herbicide tolerant Insect resistance	3 - 20	Brookes (2022)
Cotton	Insect resistance	2 – 31	Brookes and Barfoot (2017); Raman (2017); Brookes (2022)
Brinjal/Eggplant	Insect resistance	Up to 51	Ahmed et al. (2021)
Alfalfa	Reducing lignin content	15- 20	ISAAA (2017)
Canola	Herbicide tolerance	4 - 12	Brookes and Barfoot (2017)
Maize	Insect resistance Drought tolerant	5 - 24	Brookes and Barfoot (2017); Pellegrino et al. (2018); Wu et al. (2019); ISAAA (2019)
Wheat	Drought-tolerant	20 - 40	Guarin et al. (2022); Gupta (2024)
Rice	Insect resistance	Up to 28	Demont and Stein (2013)
Soybean	Herbicide tolerant, Insect resistance	3 - 20	Brookes (2022)

Furthermore, proponents claim that the advent of GM crops has helped in a new era of agricultural innovation, where crops are engineered with beneficial traits such as herbicide tolerance, insect resistance, abiotic stress tolerance, disease resistance, and nutritional enhancement (Kumar et al., 2020). With over 525 transgenic events approved for cultivation in 32 crops worldwide, the adoption of this technology has proven to be a boon for farmers and the environment alike (Kumar et al., 2020). By increasing crop yields, reducing pesticide and insecticide use, mitigating CO_2_ emissions, and lowering the cost of production, GM crops have become a cornerstone of modern agriculture (Kumar et al., 2020). Given the limitations of traditional breeding methods in meeting the demands of a fast-growing world population, it is imperative to prioritize the adoption of GM technology without compromising on their safety.

## Are foods derived from GM crops safe to human, biodiversity and environment?

Addressing concerns about GM crops requires providing trustworthy, evidence-based information. In an era of widespread misinformation, it is crucial to engage the public with clear, factual data to build trust between scientists, policymakers, and communities. This foster informed discussions on the implications of GM technology, which often raise deeper ethical and philosophical questions. Public concerns are frequently rooted in values and beliefs and addressing them respectfully can lead to more constructive conversations. While many scientists see GM technologies as a solution to global challenges, others raise concerns about human and environmental safety (Bawa and Anilakumar, 2013; Zhang et al., 2016).

Regarding safety concerns, opponents argue that there may be adverse effects on human and animal health, including allergic reactions, toxicity, harm to vital organs, gene transfer, and disparities in nutritional value (Smith-Spangler et al., 2012). Although opponents have expressed criticism and concerns, they lack substantial scientific evidence to support their arguments against the commercialization of GM products. However, proponents and risk-assessing institutions declare that these concerns stem from a lack of information and scientific misunderstanding. Proponents claim that numerous studies have been conducted to thoroughly evaluate the safety of GM crops and the foods derived from them. It has been reported that not a single study has discovered any detrimental effects of GM crops on human health (DeFrancesco, 2013). Their scientific basis is a rigorous study conducted by the US National Academy of Sciences, which concluded that GM crops are as safe to eat as their non-GM counterparts, with no documented adverse health effects (NAS, 2016). Additionally, they highlight that the European Commission (EC) has funded 130 research projects carried out by over 500 independent teams, on the safety of GM crops, and none of these studies have found any special risks associated with GM crops (Freedman, 2013). Similarly, “decade of EU-funded GMO research (2001- 2010)” project focusing on the environmental impacts, food safety, risk assessment, and management of GM crops showed that these crops are not different from the convectional counterparts (EU, 2010).

Furthermore, proponents emphasize that GM crops undergo thorough testing and evaluation before they are approved for commercialization and human consumption and their safety is equivalent or better than conventional breeds. The endorsement of GM crops has received unequivocal support from prestigious organizations such as the American Association for the Advancement of Science, the American Medical Association, and the National Academy of Sciences. Extensive evaluations conducted by the U.S. Food and Drug Administration, as well as similar regulatory bodies in various nations, have consistently affirmed that GM crops do not pose any distinct risks to human health (Freedman, 2013). According to a comprehensive review of 32 studies, spanning more than two decades of experimental trials involving GM maize, there was a notable rise in crop yield with no discernible variance in the levels of proteins, lipids, acid detergent fiber, neutral detergent fiber, and total dietary fiber in the grain when compared to isolines or near isolines (Pellegrino et al., 2018).

Based on numerous articles to reconcile the differing opinions surrounding the safety concerns of GM foods, we firmly claim that GM crops currently on the market are equally, if not more, safe than conventional varieties due to the extensive risk evaluation they undergo. Despite the lack of evidence supporting concerns about GM foods and feeds, the implementation of a precautionary assessment for foods derived from GM crops before they are made available to the public is necessary. These will not only address any potential future concerns associated with GM foods but also demonstrate to the public the high level of analysis and assessment of GM-derived foods go through. In our view, the presence of critics is essential for the progress and precision of GM technology. Without critics and concerns, the numerous technological breakthroughs that humans have achieved would not have been possible. We also believe it is important to address the concerns of those who oppose. According to Principle 15 of the 1992 Rio Declaration on Environment and Development, “In order to protect the environment, the precautionary approach shall be widely applied by States according to their capabilities.” To manage the uncertainties surrounding GMOs, the Cartagena Protocol on Biosafety to the Convention on Biological Diversity was signed by multiple parties, establishing guidelines to ensure the safe introduction, transboundary movement, handling, and use of GMO (Cartagena Protocol on Biosafety, 2000).

### Concerns about antibiotic resistance

Another concern regarding GM crops is the potential transfer of antibiotic resistance marker genes from transgenic food to gut bacteria, facilitating the spread of additional antibiotic resistance genes. This concern stems from the fact that scientists often utilize antibiotic resistance genes, such as kanamycin and hygromycin to differentiate between transformed and untransformed lines. Advocates for GM crops argue that the antibiotic resistance markers employed in genetic modification have minimal to no therapeutic impact on humans or animals (Kiran and Pandey, 2020). However, this viewpoint does not guarantee that antibiotic resistance genes are risk-free, and hence require solid evidence as consumers need to know details of food they consume.

The concern regarding antibiotic resistance has prompted researchers to innovate and devise alternative cloning strategies that do not rely on antibiotics for transformation. Scientists have begun to implement various scorable marker systems, including fluorescent proteins and enzymes. Notable examples of these markers are green fluorescent protein (GFP) (Tsien, 1998), yellow fluorescent protein (YFP) (Nagai et al., 2002), luciferase (LUC) (Smale, 2010), and β-glucuronidase (GUS) (Jefferson et al., 1987). These scorable markers offer a visual indication, such as fluorescence or color change, to identify the presence of a transgene without imposing selection pressure.

While scorable markers are considered a safer alternative to selectable markers, many consumers remain concerned about any DNA markers used for tracking their food. To address these concerns, scientists have developed marker-free transformation technologies that eliminate markers immediately after confirming successful transformation, utilizing techniques such as Cre/lox specific recombination sites (Russell et al., 1992; Hoa et al., 2002). In recent years scientists have turned to GMO-free CRISPR/Cas9 technology, which has emerged as a powerful genome-editing tool. It is evident that public concerns and the arguments of opponents have driven advancements in genetic manipulation, making it more precise and targeted. We believe that fostering public acceptance of GM technologies requires clear, transparent, and targeted communication about the use of genetic material in transformation processes and the methods employed for selecting transformants. While the risk of transferring antibiotic resistance genes from transgenic plants claimed to be minimal, it is important to take additional precautions to further reduce the risk.

### Allergic reaction and cancer concern

There is a concern that genetic engineering of food triggers allergenicity. Critics point to a case where a gene from Brazil nuts was inserted into soybeans, resulting in severe allergic reactions among individuals sensitive to nut proteins during early stage (Nordlee et al., 1996). They warn that such modifications could lead to the emergence of numerous new allergic responses. However, supporters of GM crops claim as such risk is minimal when genetic alterations do not induce the production of allergens (Noteborn et al., 2000). To be on the safer side, WHO advises genetic engineers to refrain from using DNA derived from known allergens unless they can demonstrate that the resulting proteins are non-allergenic (WHO, 2014). We believe extensive case by case assessment of GM foods can possibly avoid the possibility of harm to consumers, with food allergies before they brought to market.

The WHO International Agency on Research for Cancer has determined as glyphosate can be a probable human carcinogen (Abrams et al., 2024), which seek case-by-case evaluation and better response. On the other hand, the proponents of the technology argues as the development of insect-resistant crop varieties has a noticeable potential in the reduction cancer rates (Smyth, 2019). Typical example is mycotoxins which is both toxic and carcinogenic to humans and animals but there was a report that Bt maize contained lower concentrations of mycotoxins (29%), fumonisins (31%) and thricotecens (37%) (Pellegrino et al., 2018) when compared with the conventional ones. Other claim as genetically engineered microorganisms can be considered promising candidates for adjunctive treatment of diseases and cancers (Ari et al., 2024). Here, the focus should be the maximization of benefits and reduction of any possible and probable side effects in a way the GM crops can be much better than conventional breeds.

### Environmental concern

Anti-GMO advocates have raised concerns about the potential risks of genetic modification, such as genetic contamination, harm to non-target insect populations, herbicide-resistant weeds, and gene flow (Carreira et al., 2024). They fear genetic engineering may increase naturally occurring toxins, create new harmful compounds, and lead to greater accumulation of environmental pollutants like pesticides and heavy metals, however, do not support these claims. For example, over 89,000 farm surveys over 17 years found that insect-resistant maize reduced organophosphate and pyrethroid insecticide use, lowering toxicity exposure (Perry and Moschini, 2020). In China, insecticide use against bollworms dropped between 1997 and 2007 (Jikun et al., 2010), and research on glyphosate-resistant soybeans found they were more environmentally sustainable than non-GM varieties (Nelson and Bullock, 2003). In India, Bt cotton reduced pesticide use by 60%, lowering pesticide poisoning among farmers (Kouser and Qaim, 2011).

Proponents also argue that GM crops like Bt cotton and maize have led to fewer pesticide-related health issues (Smyth, 2019), with a medical study showing that exposure to insecticides from Bt crops resulted in better health outcomes compared to non-Bt cotton (Smyth, 2020). In addition containment at the initial stage of research (Partner, 2015) and confined field trials (CFTs) are devised as key components in the research and development process for GM crops for regulation and monitoring of the trait efficacy and safety before its deregulation in different country (Smets and Rüdelsheim, 2018). Concerns regarding gene flow have prompted scientists to explore organelles like chloroplasts, plastids, and mitochondria (Li et al., 2021) for genetic transformation, which effectively prevents gene flow.

### Loss of biodiversity concern

The debate over the impact of GM crops on biodiversity is another concern within the Convention on Biological Diversity. Critics argue that GM crops may disrupt ecosystems by allowing more dominant species to outcompete native ones and promote gene flow (Conner et al., 2003). This point of view seek better consideration as farmers usually stick to improved varieties whether it is conventional breed or GM crops. In contrast, supporters of biotech crops argue that there is no strong evidence linking GM crops to adverse effects on biodiversity, suggesting that agricultural intensification and pesticide use are the main drivers of biodiversity loss (Schütte et al., 2017). They claim as GM crops can positively affect biodiversity by reducing insecticide use, promoting eco-friendly herbicides, and enhancing agricultural sustainability (Brookes and Barfoot, 2018). According to proponents, studies found as non-target invertebrates were generally more abundant GM crop fields when compared to non-transgenic fields treated with insecticides (Marvier et al., 2007; Meissle et al., 2022). A recent study by Engist et al. (2024) found that GM crops positively influence bird populations. Furthermore, advocates argue that increased crop yields can lessen the need for land clearing for agriculture (Carpenter, 2011). There is also a claim that GM crops can help reduce agricultural greenhouse gas (GHG) emissions by up to 7.5% of European continent’s total agricultural emissions (Kovak and Blaustein-rejto, 2022). However, ongoing research is vital to thoroughly assess the long-term effects of GM crop use on deforestation and biodiversity (Noack et al., 2024). Establishing a strong monitoring system is essential to address these issues and ensure proper oversight.

### Socio-economic concerns

It has been observed that GM crops can boost agricultural yields by at least 20%. However, trade barriers on these crops have led to restricted food access, diminished farm revenues, and escalated prices, resulting in significant socio-economic challenges such as rising food costs, increased poverty, and unnecessary hardship (English, 2021). Higher yields translate to greater supply volumes, and an abundant supply is crucial for keeping food prices low. When countries remove trade barriers, it is projected that imports could rise by approximately 14.7%, leading to an estimated 4.86% decrease in food prices. In contrast, trade barriers can reduce import access by nearly 10%, causing food prices to increase by 1% (Nes et al., 2021).

Despite concerns that GM crops may not benefit smallholder farmers and could worsen social and economic conditions; numerous economic studies present a different narrative. Research on the economic effects of Bt cotton in India indicates that this it has resulted in a 24% increase in cotton yield per acre due to reduced pest damage, alongside a 50% increase in profits for smallholder farmers (Kathage and Qaim, 2012). Opponents of GMOs argue that these crops primarily benefit large agribusinesses rather than small farmers. However, proponents counter this claim by highlighting that India, the world’s largest producer of Bt cotton, predominantly cultivates this crop among smallholder farmers (Choudhary and Gaur, 2010). In India, the introduction of Bt cotton has played a role in alleviating poverty, increasing household incomes, and fostering rural development, particularly for vulnerable farmers (Subramanian and Qaim, 2010). Furthermore, a meta-analysis of the effects of genetically modified crops revealed a 22% increase in yields, a 37% reduction in chemical pesticide use, and a 68% rise in farmer profits (Klumper and Qaim, 2014). Similarly, the adoption of genetically modified eggplant in Bangladesh resulted in a 51% increase in yields and a 37.5% decrease in pesticide costs (Ahmed et al., 2020).

Many African governments’ responses to the complex issues surrounding the development and implementation of modern genetic engineering are marked by uncertainty and confusion. These challenges span social, ethical, environmental, trade, and economic concerns. The article in The Economist, titled “Better dead than GM-fed? Europe’s greens are helping to keep Africans hungry,” reinforced the mistrust many Africans feel toward this technology. The lack of a unified African stance and a strategic framework to tackle these emerging biotechnological issues has left the door open for various interest groups to take advantage of the policy uncertainty, regardless of the actual situation in Africa.

Wesseler et al. (2017) highlighted the significant costs associated with delays in adopting crucial agricultural innovations in Sub-Saharan Africa. For example, they estimated that a one-year delay in approving pod-borer resistant cowpea in Nigeria could lead to losses ranging from $33 million to $46 million, and potentially cost between 100 and 3,000 lives. Similarly, had Kenya adopted genetically modified (GM) maize in 2006, it could have saved between 440 and 4,000 lives. In 2007, Uganda had the chance to introduce a GM black sigatoka-resistant banana, which could have saved up to 5,500 lives over the past decade by protecting banana crops and improving food security. These examples highlight the significant human and economic toll of postponing the adoption of critical agricultural technologies in the region.

A world without hunger is possible, but it requires sustainable food production and distribution (Zaidi et al., 2019). Improved crop varieties could help achieve the UN’s zero hunger goal if managed properly. However, pests and diseases still pose major challenges, causing economic losses and threatening food security worldwide (Savary et al., 2019). To feed a growing population, we need to breed better crop varieties quickly using modern technologies (Hickey et al., 2019). While these technologies offer clear benefits, overregulation and public misconceptions can slow progress. Europe is particularly affected, but developing regions like Africa and Asia—where they could have the biggest impact—are also hindered (Qaim, 2020). Overly strict regulations stifle innovation, leading to fewer investments and fewer new products (Brookes and Smyth, 2024).

GM foods, if managed well, can address hunger, improve nutrition, and reduce environmental harm by increasing crop yields and cutting pesticide use (Bawa and Anilakumar, 2013). Yet, despite the success of genetic engineering in medicine, GM crops face strong opposition. This raises important questions: Why was the Green Revolution accepted, but GM crops are still controversial? Why is genetic engineering embraced in medicine but not in agriculture? We believe a global, unified biosafety framework is needed to help move agricultural technologies across borders and avoid political obstacles. This is especially important in developing nations, where GMOs are sometimes used without full understanding. **Table 2** shows global overview of GM crop on farmers field, public perception, and awareness among producers.

**Table 2:** Global overview of approved GM crop on farmers field, public perception, and awareness.

Continent	Country	Area planted (MH)	Approved crops on farm	Public perception and awareness across continent	References
North America	USA	74.4	Alfalfa, Canola Cotton, Maize Soybean, Sugar beet	Better perception and awareness due to long history of production and consumption	Woźniak-Gientka et al. (2022); ISAAA (2024); AgbioInvestor (2024)
	Canada	11.5	Canola, Maize, Soybean, Sugar beet		
Central and South America	Brazil	66.9	Cotton, Maize Soybean, Sugarcane	Varies among countries: positive in Brazil, Argentina, and Honduras; limited awareness and caution in Paraguay and Bolivia; mixed views and debates in Uruguay, Colombia, Chile, and Mexico.	Macall et al. (2020); Benitez Candia et al. (2021); Woźniak-Gientka et al. (2022); Tadich and Escobar-Aguirre (2022); ISAAA (2024); AgbioInvestor (2024)
	Argentina	23.1	Cotton, Maize, Wheat, Soybean		
	Paraguay	4.3	Cotton, Maize Soybean		
	Bolivia	1.5	Soybean		
	Uruguay	1.2	Maize, Soybean		
	Colombia	0.2	Cotton, Maize		
	Honduras	0.1	Maize		
	Chile	0.01	Canola, Maize Soybean		
	Mexico	0.01	Cotton		
Asia	India	12.1	Cotton	Awareness and perception vary. India and Myanmar have mixed views. China, Philippines, and Indonesia show growing acceptance. Awareness is limited in Pakistan,Vietnam, and Bangladesh.	ISAAA (2024); AgbioInvestor (2024); Naveen and Sontakke (2024); Woźniak-Gientka et al. (2022); Sendhil et al. (2022); Siddiqui et al. (2022)
	China	2.8	Cotton		
	Pakistan	2.3	Cotton		
	Philippines	0.6	Maize, Rice		
	Vietnam	0.2	Maize		
	Myanmar	0.1	Cotton		
	Indonesia	0.02	Maize, Sugarcane		
	Bangladesh	0.003	Brinjal/Eggplant		
Africa	South Africa	3.3	Cotton, Maize Soybean	Low levels of awareness and perception due to misinformation and significant debate.	Kedisso et al. (2022); Masehela and Barros (2023); Rabuma et al. (2024); ISAAA (2024); AgbioInvestor (2024); Sadikiel Mmbando (2024); Catherine et al. (2024)
	Sudan	0.196	Cotton		
	Ethiopia	0.008	Cotton		
	Kenya	0.005	Cotton		
Europe	Spain	0.0463	Maize	Most of the European Union is reluctant to accept GM crops, with consumers generally having a negative perception and significant debate.	Turnbull et al. (2021); Woźniak-Gientka et al. (2022); ISAAA (2024); AgbioInvestor (2024)
	Portugal	0.0017	Maize		
Oceania	Australia	1.4	Canola, Cotton Safflower, Alfalfa	Farmers are aware and interested in cultivating GM crops if they are safe and affordable.	

## Does terminator technology affect the interest of producers?

Terminator technology, also called genetic use restriction technology (GURT) or suicide seeds, is a way to control the use of GM crops by turning on or off specific genes in response to certain triggers, like making second-generation seeds that are unable to reproduce (Swanson and Goschl, 2000). Advocates argue that terminator seeds can prevent cross-pollination with non-GMO crops, thereby safeguarding the purity of traditional varieties (Rakshit, 1998). This is particularly advantageous for farmers who wish to protect their crops from contamination. From this perspective, terminator technology plays a vital role in preserving biodiversity and safeguarding natural ecosystems, especially when concerns arise regarding gene flow in genetically modified crops. Nevertheless, there are significant ethical concerns surrounding terminator technology, as it can produce seeds that are genetically programmed to be sterile after the first generation.

Critics argue that the implementation of terminator technology may lead to an increased reliance on seed companies, restricting farmers from saving seeds for future planting (Gull et al., 2022; Caradus, 2023). Additionally, opponents highlight concerns about the potential monopolization of the seed industry by large corporations, potentially resulting in greater control over global food production. We believe that it is imperative to consider both the possible advantages and disadvantages associated with the utilization of terminator technology by food and corporate entities. Companies should not solely concentrate on generating seeds for profit but should also emphasize the importance of establishing a strong infrastructure that enables developers to capitalize on their advancements. If we consider drug manufacturers, they are beneficiaries due to their constant production of various medications. In the absence of a similar system to benefit seed-producing companies, it is unlikely to come across corporate producers operating without any form of profit. The ongoing discussion on terminator technology requires a thorough examination of its long-term implications and a balanced assessment of its pros and cons. A proponent of the technology argues that the technology is only a protection system with no threat to the patent system. In addition to this, many farmers have experience of using hybrid seed technology where they will not be going to save the seed for the next season as the technology use the advantage of heterosis between two diverse genotypes to achieve maximum hybrid vigor (Chakrabarty et al., 2023). We believe that there must exist a shared comprehension between scientists and farmers concerning the imperative nature of employing TT. Given that corporations create TT seeds with the aim of optimizing profits, these profits can be obtained by means of intellectual property rights (IPRs) that have restricted durations.

## Intellectual property right of GM crops

The primary aim of IPRs is to achieve a balance between the exclusive rights granted to inventors and the benefits the society gains from their innovations (Adcock, 2007). In the context of GM crops, seed developer companies often secure patents for specific innovations, granting them exclusive rights to these sequences for a period of 15 to 20 years in the United States. This period of exclusivity is designed to incentivize innovation by allowing inventors time to commercially develop their products (McDougall, 2011; Solanki and Chauhan, 2019). Seed companies argue as IPRs can provide a mechanism for securing returns on investment, often through royalties or partnerships, thus ensuring continued R&D funding for future advancements. Furthermore, developers believe as IPRs can incentivize innovation in agriculture and food production by providing exclusive rights and potential financial rewards to inventors and developers (Chinwe et al., 2018; Amentae et al., 2024). It is argued that the use of IPRs systems in plant breeding is essential for promoting the deployment of good agricultural practices (Smulders et al., 2021; Fuglie and MacDonald, 2023).

The debate around patent ownership by large companies often raises concerns that small breeding companies and farmers are restricted in improving or using patented crop varieties (Smulders et al., 2021). Critics argue that IPRs for GM crops create barriers to access and limit the choices available to farmers (Karampaxoglou, 2015; Wynberg et al., 2021). Critics also highlight ethical issues like high crop costs, dependency, and market manipulation (Karampaxoglou, 2015). According to this line of argument, when farmers purchase GM seeds, they may face certain restrictions and obligations imposed by the seed developers. This can restrict their freedom to save and exchange seeds, which has traditionally been an integral part of farming practices. Additionally, there are concerns for small-scale producers, particularly African farmers. Consequently, some individuals believe that IPR in African agriculture are detrimental to local food production and small-scale farming systems (Kuyek, 2002; Beumer and Swart, 2021). Kerle (2007) argued that there should be standardized effective GM crop protection that would lead to more investment in private R&D. She suggested an international obligatory standards mandating reasonably strong and harmonized GM crops patents are most likely necessary. However, policy options exist to prevent excessive concentration of power, such as the research exemption, which allows breeders to use protected varieties for research (Adcock, 2007). Developing countries can also access innovations through humanitarian licenses (Qaim and Traxler, 2005).

In more pronounced way, Heydari and Razmkhah (2014) claim as IPRs could have a detrimental effect on the right to work for vulnerable farmers limiting the role of farmers in developing countries. In these contexts, IPRs should not focus on privatization and control but should instead empower local communities, farmers, and indigenous peoples, recognizing their long-standing contributions to conserving and enhancing biodiversity for the benefit of humanity (Kuyek, 2002). There is always growing call for policy reforms aimed at making the distribution of IP-related benefits more equitable and accessible.

We believe establishing fairness between the breeder and the farmer is a precondition for realizing trade and stimulating innovation. We hold the belief that the integer part of IPRs can be advantageous for corporate companies. We also share the fact that high investment is required to develop new GM technologies and products, stronger intellectual property protection is necessary to stimulate research and to allow recovery of investment. Moreover, we emphasize that companies, apart from obtaining benefits, should also prioritize the benefits of small-scale farmers, such as offering seeds at affordable prices. Additionally, it is imperative that the duration of IPRs is not viewed as a mere period of monopoly to solely obtain benefits. As there are conflicting views with limited empirical research on IPRs, further comprehensive empirical investigations are required to understand the subject and better inform policymakers in developing countries. It is important to understand the rationale behind IPRs before engaging ethical issues surrounding IPRs (Adcock, 2007). The regulations governing this matter should be robust enough that does not significantly compromise the benefits to producers. However, we believe there should be a win-win situation so that companies are not discouraged from engaging in the costly process of innovation.

## Is GMO Labeling important?

The debate over GMO labeling has been ongoing for years, with some arguing that consumers have a right to know what is in their food, while others claim that such labeling is unnecessary. GM food labeling regulations vary among countries (Smith, 2018). In the United States, Food and Drug Administration (FDA) does not require labeling of GM foods unless they are significantly different from their conventional counterparts in terms of nutritional content or potential allergenicity (Whitman, 2000). On the other hand, the European Union (EU) has implemented strict regulations that mandate the labeling of all GM foods, regardless of any perceived differences (Whitman, 2000). These differences in labeling requirements reflect the varying attitudes and perception towards GM foods in different countries. While the US focuses on the safety and substantial equivalence of GM foods, the EU argues to prioritize consumer choice. This divergence in regulations has led to trade disputes between the US and the EU, as well as debates about the transparency and adequacy of GM food labeling worldwide. Some scholars argue that mandatory labeling of GMOs can improve attitudes toward genetically engineered food by eliminating buyer’s confusion (Kolodinsky and Lusk, 2018; Ryan et al., 2024). It has been reported that labeling policy had led to a 19% reduction in opposition to GE food and positive effects on the consumers were observed for reduced price and perceived quality (Piton et al., 2020).

One group of thought argues that labeling GM foods enables consumers to make well-informed decisions regarding their purchases and consumption. This level of transparency allows individuals to align their dietary preferences with their values, whether it involves supporting sustainable agriculture or avoiding specific allergens (Oh and Ezezika, 2014; Messer et al., 2017). Additionally, labeling GM foods can be beneficial for individuals with dietary restrictions or allergies, aiding them in identifying and steering clear of products containing genetically modified ingredients that may trigger adverse reactions. Moreover, individuals adhering to religious or cultural dietary guidelines can utilize labeling to select products that align with their beliefs (Oh and Ezezika, 2014; Hu et al., 2005). Furthermore, the labeling of GM foods can contribute to enhancing overall food safety and fostering consumer trust in the food industry (McCluskey et al., 2018; Zheng and Wang, 2021). By disclosing the presence of genetically modified ingredients, consumers can have confidence in the regulatory processes and the transparency of food producers. This transparency helps build trust between consumers and manufacturers, thereby promoting a healthier and more sustainable food system.

Another group argues labeling GM products may negatively impact the agricultural industry and the public perception of GM foods. Critics suggest that labeling could lead to a stigma against GM foods, causing consumers to avoid them altogether (Oh and Ezezika, 2014; Goodman, 2024). This group of thought expresses concerns that stigmatization may hinder further research and development in genetic engineering, potentially limiting advancements in agriculture. In addition they question the rational of call for generic labeling of GM food as there were no any cases where post-market surveillance has uncovered harm to consumers or the environment including potential transfer of DNA from the GMO to non-target organisms since its first approval for commercial production in 1995 (Goodman and Goodman, 2024). In our opinion, it is crucial to consider the preferences of most consumers when it comes to labeling. We contend that if food items obtained from genetically modified crops are proven to be safe for consumption, labeling will not negatively impact the commercialization and utilization of GM crops. If GM crops are as safe as or even safer than their organic or conventional counterparts, we believe that labeling should not be a concern in the future.

## Conclusion

Despite the significant increase in global cultivation of GM crops and the foods derived from them, there continue to be debates between the proponents and opponents. The debate between both sides is intense and requires a harmonized approach to harness this technology for societal benefits. This paper emphasizes the importance of finding common ground between the arguments of both opponents and proponents, while also presenting our own viewpoints. We argue that labeling GM crops as unnatural is an unsubstantiated claim lacking scientific backing. Likewise, characterizing GM technology as analogous to playing God is inappropriate and driven by baseless animosity. Furthermore, the claim that GM crops do not enhance yield is not based on strong evidence. Moreover, the claim that foods originating from GM crops are unsafe for consumption is not justified. We have also underscored the need to have intellectual property rights that benefit seed developers within limited terms without monopolizing farmers. Finally, we advocate for the labeling of foods derived from GM crops as we believe it can enhance transparency and consumer choice.
